# Epidermal growth factor promotes cyclin G2 degradation via calpain-mediated proteolysis in gynaecological cancer cells

**DOI:** 10.1371/journal.pone.0179906

**Published:** 2017-06-22

**Authors:** Stefanie Bernaudo, Shahin Khazai, Eilyad Honarparvar, Alina Kopteva, Chun Peng

**Affiliations:** 1 Department of Biology, York University, Toronto, Canada; 2 Centre for Research on Biomolecular Interactions, Faculty of Science, York University, Toronto, Canada; Florida State University, UNITED STATES

## Abstract

Cyclin G2 (CCNG2) is an atypical cyclin that functions to inhibit cell cycle progression and is often dysregulated in human cancers. We have previously shown that cyclin G2 is highly unstable and can be degraded through the ubiquitin/proteasome pathway. Furthermore, cyclin G2 contains a PEST domain, which has been suggested to act as a signal for degradation by multiple proteases. In this study, we determined if calpains, a family of calcium-dependent proteases, are also involved in cyclin G2 degradation. The addition of calpain inhibitors or silencing of calpain expression by siRNAs strongly enhanced cyclin G2 levels. On the other hand, incubation of cell lysates with purified calpains or increasing the intracellular calcium concentration resulted in a decrease in cyclin G2 levels. Interestingly, the effect of calpain was found to be dependent on the phosphorylation of cyclin G2. Using a kinase inhibitor library, we found that Epidermal Growth Factor (EGF) Receptor is involved in cyclin G2 degradation and treatment with its ligand, EGF, induced cyclin G2 degradation. In addition, the presence of the PEST domain is necessary for calpain and EGF action. When the PEST domain was completely removed, calpain or EGF treatment failed to trigger degradation of cyclin G2. Taken together, these novel findings demonstrate that EGF-induced, calpain-mediated proteolysis contributes to the rapid destruction of cyclin G2 and that the PEST domain is critical for EGF/calpain actions.

## Introduction

Cyclins encompass a group of closely related molecules. Classical cyclins accumulate periodically to activate their associated cyclin-dependant kinases (Cdk) and stimulate the mitotic events that regulate the rate of cell division [1]. The concentration of cyclins oscillate during the cell cycle [2], which allows for the strong unidirectional flow of cellular division. Cyclin expression levels are regulated by highly orchestrated protein turnover events and many cyclins are targeted for rapid degradation due to the presence of a destruction box or a PEST domain [3–5]. The Ubiquitin-Proteasome Pathway (UPP) is critical for the degradation of many short-lived proteins; and all known cyclins are targeted by the UPP [2, 3, 6, 7].

Cyclin G2 belongs to a group of unconventional cyclins that includes cyclin G1 and cyclin I [8, 9]. Unlike typical cyclins that promote cell cycle progression, cyclin G2 functions to maintain the quiescent state of cells via the induction of cell cycle arrest [10–12]. Cyclin G2 contains distinct features that imply its temporal level and strict regulation throughout the cell cycle. Firstly, a destabilizing domain, PEST, which controls the stability of many proteins, is located at the C-terminal end; and secondly, a cyclin box, structurally similar to cyclin A, is centrally located within the protein [9]. Increasing evidence suggests that cyclin G2 acts as a tumor suppressor. Indeed, overexpression of cyclin G2 inhibits proliferation in many cell lines [12–14], and an inverse relationship is observed between cyclin G2 levels and cancer progression [10, 15, 16]. In addition, there exists a significant difference in the level of cyclin G2 in normal versus malignant tissues [15–18]. Furthermore, growth inhibitory signals promote [19–22], while oncogenic pathways reduce [10, 23–25] cyclin G2 levels. We recently demonstrated that cyclin G2 exerts tumor-suppressive effects by inhibiting epithelial-to-mesenchymal transition (EMT) via suppression of Wnt/ß-catenin signaling in ovarian cancer cells [18].

Calpains are a family of intracellular, calcium-activated, cysteine proteases that are divided into two main groups: calpain-1 (μ-calpain) and calpain-2 (m-calpain) [26]. They are ubiquitously distributed throughout all cells, and play important and diverse roles in physiology and pathology [26, 27]. Both isoforms have similar biochemical characteristics and differ mainly in the amount of calcium needed for their activation. Calpain-1 requires *in vitro* calcium levels in the micromolar range, whereas calpain-2 is activated when calcium levels are in the milimolar range [27]. The end result of calpain activity is usually not simple destruction, but more often results in alteration of the target protein in a limited proteolytic manner. Under these conditions, the protein may become active, inactive or more susceptible to other digestive pathways [26, 27].

We have previously demonstrated that cyclin G2 is highly unstable and treatment with a proteasome and calpain inhibitor, MG-132, strongly enhanced cyclin G2 stability in an immortalized ovarian epithelial cell line and in OV2008 [28]. We further showed that cyclin G2 can be degraded quickly by the UPP. Since OV2008 has recently been reclassified as a cervical cancer cell line [29], we assessed cyclin G2 stability in several ovarian cancer cell lines. We also investigated if cyclin G2 can be degraded by calpains, and if this process is dependent on prior phosphorylation and/or the presence of the PEST domain. Through screening of a kinase inhibitor library, we identified the EGF Receptor (EGFR) as an activator of cyclin G2 degradation and demonstrated that EGF reduced cyclin G2 stability via a calpain-mediated pathway.

## Methods and materials

### Chemicals and reagents

M2 anti-FLAG antibody, cyclohexamide (CHX), and CKII inhibitor, DMAT, were purchased from Sigma-Aldrich (Oakville, ON, Canada). Recombinant human EGF and V5 antibody were from Life Technologies (Burlington, ON, Canada). Anti-p-Tyr, anti-GAPDH and anti-β-actin were purchased from Santa Cruz Biotechnology (Santa Cruz, CA, USA) and used to detect non-specific phosphorylated tyrosine residues. Purified calpains, calpeptin, N-Ac-Leu-Leu-norleucinal (ALLN), and A23187 were purchased from Calbiochem-Novabiochem (San Diego, CA, USA). A kinase inhibitor library was obtained from Enzo Life Sciences (Farmingdale, NY, USA). Calf intestinal phosphatase (CIP) was purchased from New England Biolabs (Whitby, ON, Canada). GF109203X and NU 112455A were from Selleckchem (Houston, TX) and EMD Millipore (Etobicoke, ON), respectively.

### Cell culture and cell lines

A cervical cancer cell line OV2008 was obtained and cultured as previously reported [28, 30]. Ovarian cancer cell lines, SKOV3.ip1, and ES2 cells have also been described previously [18]. To generate stable cell lines, FLAG-tagged cyclin G2 cDNA (FLAG-CCNG2) was cloned into a plasmid vector, pBabe-puro. Virus was produced in 293T cells by calcium chloride transfection of 10μg of either pBabe-FLAG-CCNG2 or pBabe-empty vector, 6.5μg VSVg and 3.5μg PUMVC for packaging. After overnight transfection, the media was changed and the virus was harvested, 6 hours later, by passing the media through a 0.45 μM filter. The medium containing the virus was immediately used to infect each target cell line. Stable cell lines were maintained in puromyocin selection media and cyclin G2 expression was confirmed by Western blot using an antibody against the FLAG tag.

### Plasmids, RNA interference, and transfection

The FLAG-CCNG2 and CCNG2-V5 (WT, PEST24, and ΔPEST) plasmids were generated as described previously [28]. Transient transfection was carried out using Lipofectamine 2000 (Life Technologies) according to the manufacturer’s protocol. Small interference RNA (siRNA, 50 nm) was transfected into cells for 6 h using Lipofectamine 2000. Calpain-1, calpain-2, and a scrambled control siRNA, which has no significant homology to any mammalian gene sequence, were purchased from GenePharma (Shanghai, China): Calpain-1 siRNA: 5-AAACUAGCUGGCAUCUUCTT-3; Calpain-2 siRNA: 5-GAAGUGGAAACUCACCAAATT-3; and Control siRNA: 5-UUCUCCGAACGUGUCACGUTT-3.

### Treatment with kinase inhibitor library

To determine which kinase is involved in cyclin G2 degradation, cells were treated with 10 μM of the inhibitor or its vehicle DMSO for 2 hours in the presence of CHX. Cells were lysed and analyzed by Western blot using anti-FLAG antibody to determine cyclin G2 levels. The inhibitors tested are listed in S1 Table.

### Western blot analysis

Cell lysates were prepared and Western blot was performed as reported previously [31]. Briefly, cells were lysed in a buffer containing 150 mm NaCl, 1% Nonidet P-40, 50 mm Tris/HCl (pH 7.4) supplemented with protease and phosphatase inhibitors (Life Technologies), separated by 10–12% SDS-PAGE and transferred to a PVDF membrane. Cyclin G2 was detected using enhanced chemiluminesence (Luminata Classico Western HRP Substrate, EMD Milipore) and antibodies specific for either the FLAG- or V5-tag. GAPDH or β-actin was used as loading controls.

### Prediction of calpain cleavage sites and phosphorylation sites on cyclin G2

Potential calpain cleavage sites on cyclin G2 was predicted using CALPCLEAV [26]. Prediction of phosphorylation sites were performed using GPS 2.1[32] and Netphos 3.1[33].

### Calcium-induced degradation assay

SKOV3.ip1 or OV2008 cells stably transfected with FLAG-CCNG2 were lysed using a degradation lysis buffer (10mM Tris/HCl, pH 7.4, 100mM NaCl, 1mM DTT, and 0.5% Triton-X) containing either 0, 0.5, or 5mM CaCl_2_ for 60 or 120 minutes. Reactions were stopped with SDS sample buffer and analyzed by SDS/PAGE. Samples were immunoblotted with anti-FLAG for cyclin G2 expression.

OV2008 cells were transfected with either full-length CCNG2-V5 or PEST deletion mutants (PEST24 or ΔPEST). Following transfection, cells were pretreated with or without 20μM ALLN for 30 minutes. Cells were then treated for an additional 2 hours with either DMSO as a control, or 1μM of the calcium ionophore, A23187. Cells were lysed and analyzed by Western blotting. Cyclin G2 was detected using antibody against the V5-tag.

### *In vitro* calpain degradation assay

Cells stably transfected with FLAG-CCNG2 were lysed the degradation lysis buffer. Equal amounts of protein were separated into 1.5mL tubes and supplemented with vehicle control, CaCl_2_, Calpain-1, calpeptin, or a combination of these, as indicated. Reactions took place for 1 hour at 30°C. Samples were immediately run on SDS/PAGE gels and immunobloted for FLAG-cyclin G2 levels. In some experiments, cells were treated with CIP and an EGFR kinase inhibitor (Tryphostin AG1478) before the *in vitro* calpain degradation assay. For CIP treatment, 1 unit of CIP/μg of protein was added to the raw lysate for 30 mins at 30°C. For AG1478 treatment, 10μM of inhibitor was added to cell culture media for 30 mins prior to lysis. An additional 10μM of inhibitor was added to the raw lysate.

### Casein zymography

Zymography was used to determine the activity of calpain-1 and calpain-2. Cells were lysed with the degradation lysis buffer, mixed with one volume of sample buffer (300mM Tris, 40% glycerol, 0.02% bromophenol blue, and 100mM DTT, pH 6.8) and run immediately on a 12% acrylamide gel polymerized with casein. Following electrophoresis, gels were incubated in 5mM CaCl_2_ and 10mM DTT for 1 hour, buffer was refreshed and further incubated for 16 hours. Gels were then stained with Coomassie blue and calpain-1 and calpain-2 are visualized by band clearing.

## Results

### Cyclin G2 is degraded by calpains and the PEST domain is required for the calpain-mediated degradation

**Fig 1:**
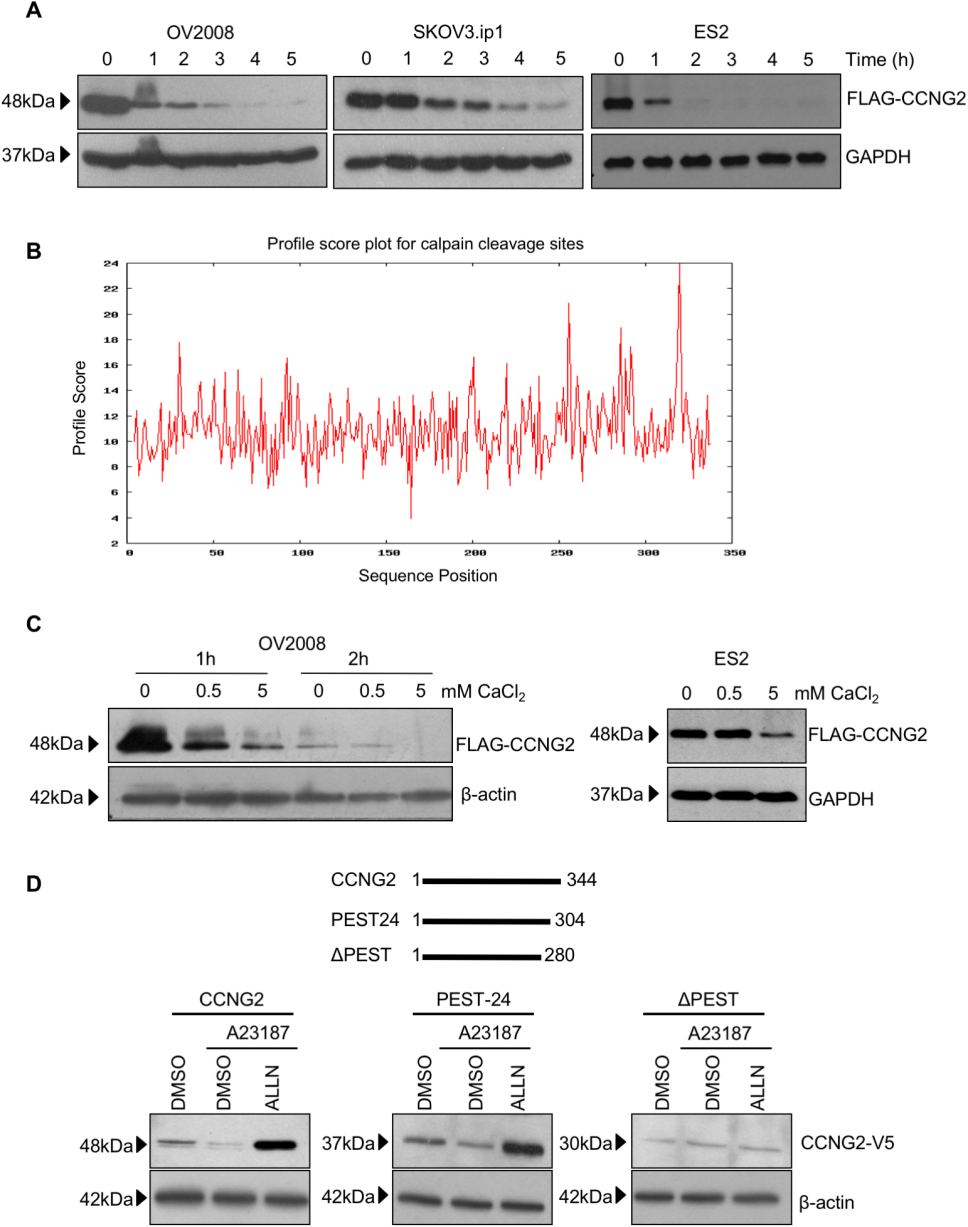
Cyclin G2 is an unstable protein and can be degraded through a calcium-mediated mechanism. A) OV2008, SKOV3.ip1, or ES2 cells stably transfected with a FLAG-tagged cyclin G2 (FLAG-CCNG2) were seeded at equal densities and treated with 10μg/ml cychoheximide (CHX) to block *de novo* protein synthesis. Cells were lysed before CHX treatment (time 0) or at 1 to 5 hour after CHX treatment. Cyclin G2 levels were determined by Western blotting using an anti-FLAG antibody. B) Prediction of calpain cleavage site using CALPCLEAV. Many potential sites were found; however, the one located in the PEST domain between position 319 and 320 had the highest score. C) Lystaes of OV2008 or ES2 cells stably transfected with FLAG-CCNG2 were incubated in a buffer containing different concentrations of CaCl_2,_ for 1–2 hours (OV2008) or 1 hour (ES2). Cyclin G2 levels were analyzed by Western blotting. Increased calcium concentration resulted in decreased amounts of cyclin G2. D) Upper panel: Schematic representation of three cyclin G2 constructs, full-length-CCNG2 (CCNG2), PEST-24 (containing the first 24 amino acid of the PEST domain), and ΔPEST (complete removal of the PEST domain). Lower panel: Cyclin G2 wild type and deletion constructs were used to transfect OV2008 cells. Following transfection, cells were treated with either DMSO as a control or 1μM of the calcium ionophore, A23187, with or without pre-incubation with 20μM ALLN for 30 minutes. Cells were lysed and Western blot analyses were performed. Treatment with A23187 decreased the levels of full length and PEST-24 cyclin G2 while the protease inhibitor, ALLN, protected cyclin G2 from degradation. A23187 and ALLN had no effect on the level of ΔPEST.

We have previously demonstrated the unstable nature of cyclin G2 in OV2008 cells [28]. However, a recent study has shown that OV2008 cells are actually of cervical cancer origin [29]. Therefore, we further tested the stability of cyclin G2 in several ovarian cancer cell lines and compared the results with OV2008. OV2008, SKOV3.ip1, and ES2 cells stably transfected with a FLAG-CCNG2 construct were seeded at equal densities and treated with CHX for 0–5 hours to block *de novo* protein synthesis. Decreases in cyclin G2 levels were observed within the first 1–2 hours following CHX addition for all cell lines tested (Fig 1A); further supporting the instability of cyclin G2.

The PEST domain, which acts as a signal for protein degradation via proteasome and/or calpain mediated pathways, is a common feature in unstable proteins and is present at the C-terminal of cyclin G2 [34, 35]. CALPCLEAV, a server that predicts the potential calpain cleavage sites along a protein sequence, found the highest probability of calpain cleavage is within the PEST domain of cyclin G2, between position 319 and 320 (Fig 1B). Therefore, we investigated if calpains play a role in the rapid destruction of the cyclin G2 protein. Since calpain activation has been shown to be dependent on calcium [36], we first tested if calcium affects the degradation of cyclin G2. Cell lysates from FLAG-cyclin G2-transfected OV2008 and ES2 were incubated in buffers containing different concentration of CaCl_2_ and Western blot analysis revealed an inversely proportional relationship between the level of cyclin G2 and the concentration of CaCl_2_ (Fig 1C). To verify that calcium plays a role in cyclin G2 stability and to determine which region on cyclin G2 the calcium-activated degradation occurs, OV2008 cells were transfected with full length, PEST-domain partial deletion (PEST-24), or PEST-domain deleted (ΔPEST) cyclin G2 constructs. The cells were then treated with a calcium ionophore, A23187, alone or in the presence of a protease inhibitor, ALLN [37]. Compared to the control, treatment with 1μM A23187 decreased the level of full-length cyclin G2, and to a less extent, the PEST24 mutant. However, it did not affect the stability of the ΔPEST mutant. Pre-incubation with ALLN abolished calcium-induced cyclin G2 degradation. Although ALLN strongly increased full cyclin G2 levels, this effect was eliminated if the entire PEST domain was removed (Fig 1D).

**Fig 2:**
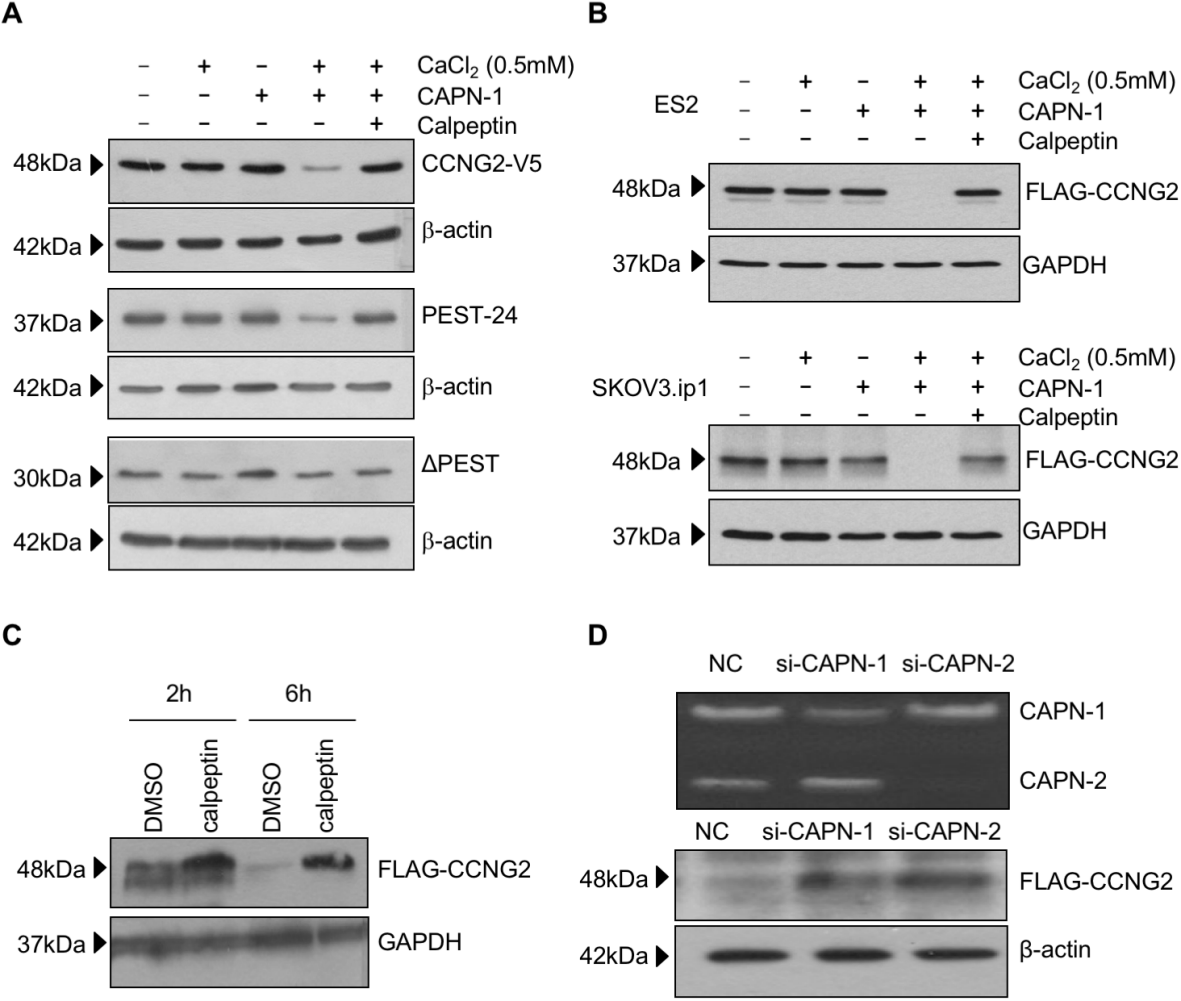
Cyclin G2 is a target of calpain-mediated proteolysis. A) OV2008 cells were transiently transfected with full-length cyclin G2 (CCNG2-V5), PEST-24, or ΔPEST and lysed in a buffer containing either calcium, purified calpain-1 (CAPN-1), or the combination of calcium and CAPN-1, with or without the calpain inhibitor, calpeptin. Calpain-1 induced the degradation of full length cyclin G2 and PEST-24 and this effect was attenuated by calpeptin. However, the level of ΔPEST was not affected by either calpain or calpeptin. B) Calpain promoted the degradation of cyclin G2 in ovarian cancer cells. ES2 and SKOV3.ip1 cells stably transfected with FLAG-CCNG2 were lysed in a buffer containing calcium, CAPN-1, and/or calpeptin, as indicated. The combination of calcium and calpain dramatically decreased cyclin G2 levels, whereas calpeptin reversed this effect. C) Inhibition of calpain activity enhanced cyclin G2 levels. OV2008 cells were transiently transfected with the FLAG-CCNG2 plasmid and treated with or without 50μM calpeptin for 2h or 6h. Western blot analysis demonstrated a protective effect of calpeptin on cyclin G2 stability. D) Silencing of calpains increased cyclin G2 levels. Top panel, OV2008 cells were transfected with siRNAs for calpain-1 (si-CAPN-1) or calpain-2 (si-CAPN-2) and casein zymography was performed to confirm the down-regulation and specificity of each siRNA for its respective calpain. Calpain-2 is identified by higher mobility on the gel. Bottom panel, OV2008 cells were transfected with si-CAPN-1 or si-CAPN-2 for 6 hours prior to overnight (16 hour) transfection of FLAG-CCNG2. Cells were recovered for 6 hours in the presence of CHX. Both calpain siRNAs increased cyclin G2 stability. NC, non-targeting control.

To determine if calpain activation is responsible for the degradation of cyclin G2, OV2008 cells, transiently transfected with CCNG2-V5 plasmids, were lysed in a non-denaturing buffer with or without calcium, calpain-1, and/or a calpain inhibitor, calpeptin. In the presence of calcium, calpain-1 strongly decreased the level of full-length cyclin G2, and to a lesser extent, PEST-24, while the effect of calpain was reversed by co-treatment with calpeptin. Removal of PEST domain abolished the effect of calpain (Fig 2A). A similar effect of calpain-1 on cyclin G2 degradation was observed in ES2 and SKOV3.ip1 cells (Fig 2B). Additionally, when CCNG2 transfected-OV2008 cells were treated with calpeptin, there was a marked increase in cyclin G2 levels at both 2 and 6 hours post treatment (Fig 2C). To further confirm the involvement of calpains in cyclin G2 degradation, siRNAs targeting calpain-1 or calpain-2 were transfected into OV2008 cells. Casein zymography confirmed that each siRNA knocked down its specific target, with no cross-reactivity to each other (Fig 2D). Knockdown of either calpain-1 or calpain-2 resulted in an accumulation of cyclin G2 protein levels, as compared to the negative control siRNA (Fig 2D).

### Phosphorylation of cyclin G2 targets the protein for calpain-mediated proteolysis

**Fig 3:**
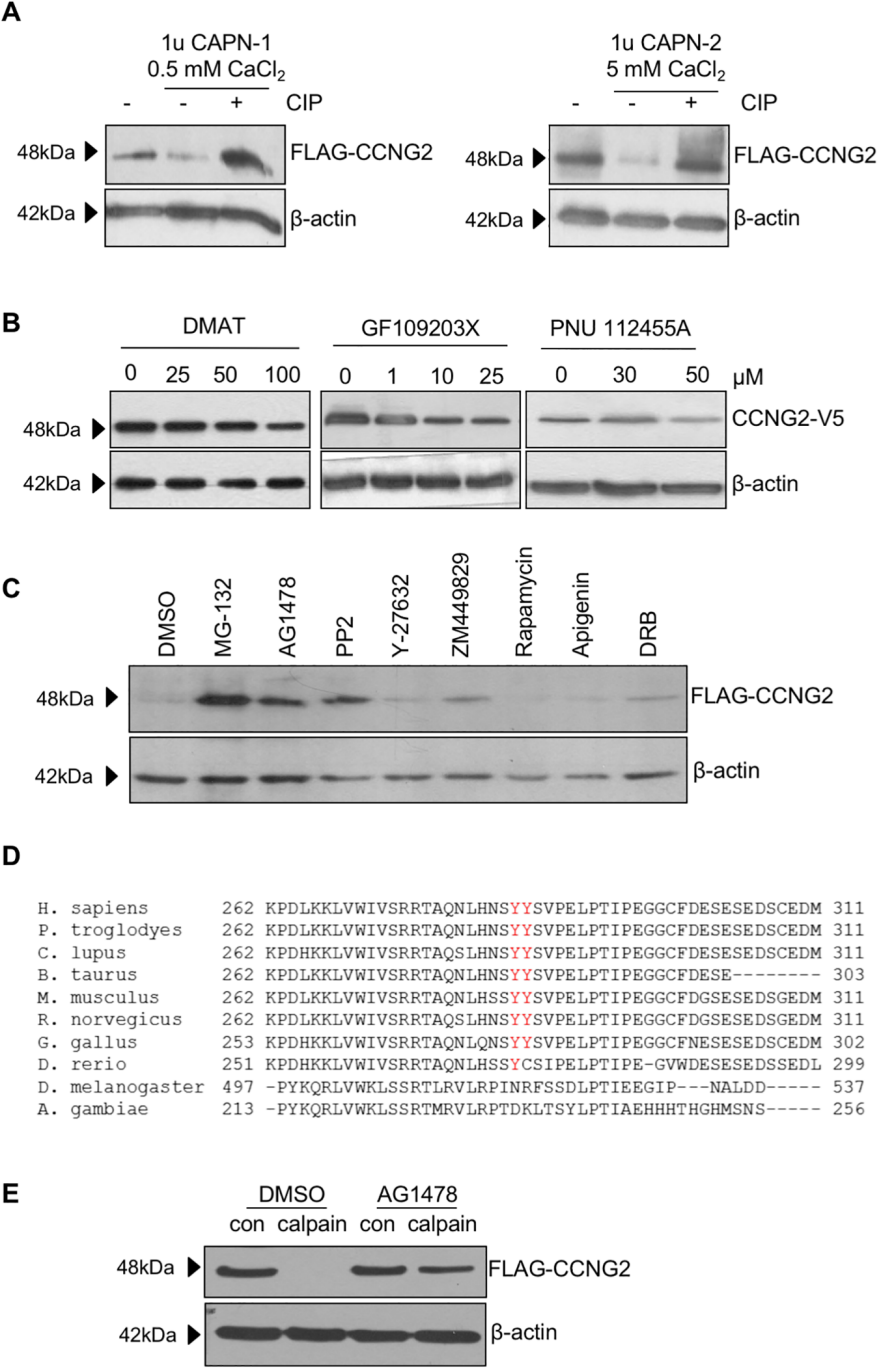
Phosphorylation is involved in calpain-mediated cyclin G2 degradation. A) OV2008 cells stably transfected with FLAG-CCNG2 were pretreated with CIP to dephosphorylate proteins and then further incubated with purified calpain-1 (CAPN-1) or calpain-2 (CAPN-2) and calcium. CIP treatment abolished the effect of calpains on cyclin G2 degradation. B) OV2008 cells were transfected with CCNG2-V5 and recovered in the presence of various concentrations of either DMAT, a CKII inhibitor, GF109203X, a PKC inhibitor, or PNU 112455A, a Cdk2/5 inhibitor. Cyclin G2 levels were analyzed by Western blot. CKII, PKC, and CDK5 inhibitors did not affect cyclin G2 stability. C) Effects of various kinase inhibitors on cyclin G2 degradation. MG-132 was used as a positive control. Western blot analysis showed that treatment with 10μM Tyrphostin AG1478, which inhibits EGFR, and PP2, which inhibits Src and EGFR, protected cyclin G2 from degradation. D) Aligned conserved residues of cyclin G2 from amino acid 262 to 311 across various species. The predicted EGFR and Src phosphorylation sites, at position 284 and 285, are well conserved in vertebrates. E) OV2008 stable cells that express cyclin G2 were treated with either DMSO or AG1478, for 30 minutes prior to lysing the cells. Whole cell lysates were further incubated with 5 mM calcium chloride and purified calpain-2 for 1 hour. Inhibition of EGFR blocked the effect of calpain on cyclin G2 degradation.

To further determine how calpains regulate the proteolysis of cyclin G2, we investigated the possibility of an upstream phosphorylation event that could label cyclin G2 as a target for activated calpains. Whole cell lysates from FLAG-CCNG2 G2 transfected OV2008 cells were pretreated with calf intestinal phosphatase (CIP) to dephosphorylate proteins, and subsequently incubated with purified calpains and calcium. Both calpain-1 and calpain-2 decreased the level of cyclin G2, however this effect was attenuated by pre-treatment with CIP, suggesting that phosphorylation of cyclin G2 is required for its proteolysis by calpains (Fig 3A).

Examination of the cyclin G2 primary sequence using bioinformatics tools revealed many potential phosphorylation sites by kinases, including CKII, PKC, and CDK5. However, treatment with inhibitors of these kinases did not protect cyclin G2 from degradation (Fig 3B). Therefore, we purchased a kinase inhibitor library to screen a variety of possible kinases. Among all the inhibitors tested (S1 Table), only Tyrphostin AG1478, which is an EGFR inhibitor, and PP1 and PP2, which are Src inhibitors but have been reported to also inhibit EGFR [38], resulted in an increase in cyclin G2 stability (S1 Table and Fig 3C). Furthermore, analysis of the amino acid sequence of cyclin G2 revealed that there are potential EGFR/Src phosphorylation sites at residues 284 and 285. These sites are highly conserved from chicken to mammal (Fig 3D). To confirm that EGFR signaling participated in calpain-mediated degradation of cyclin G2, an *in vitro* degradation assay was performed in the absence or presence of AG1478. As shown in Fig 3E, pretreatment with AG1478 attenuated the effect of calpain on cyclin G2 degradation.

### Activation of EGFR contributes to cyclin G2 degradation by calpain

**Fig 4:**
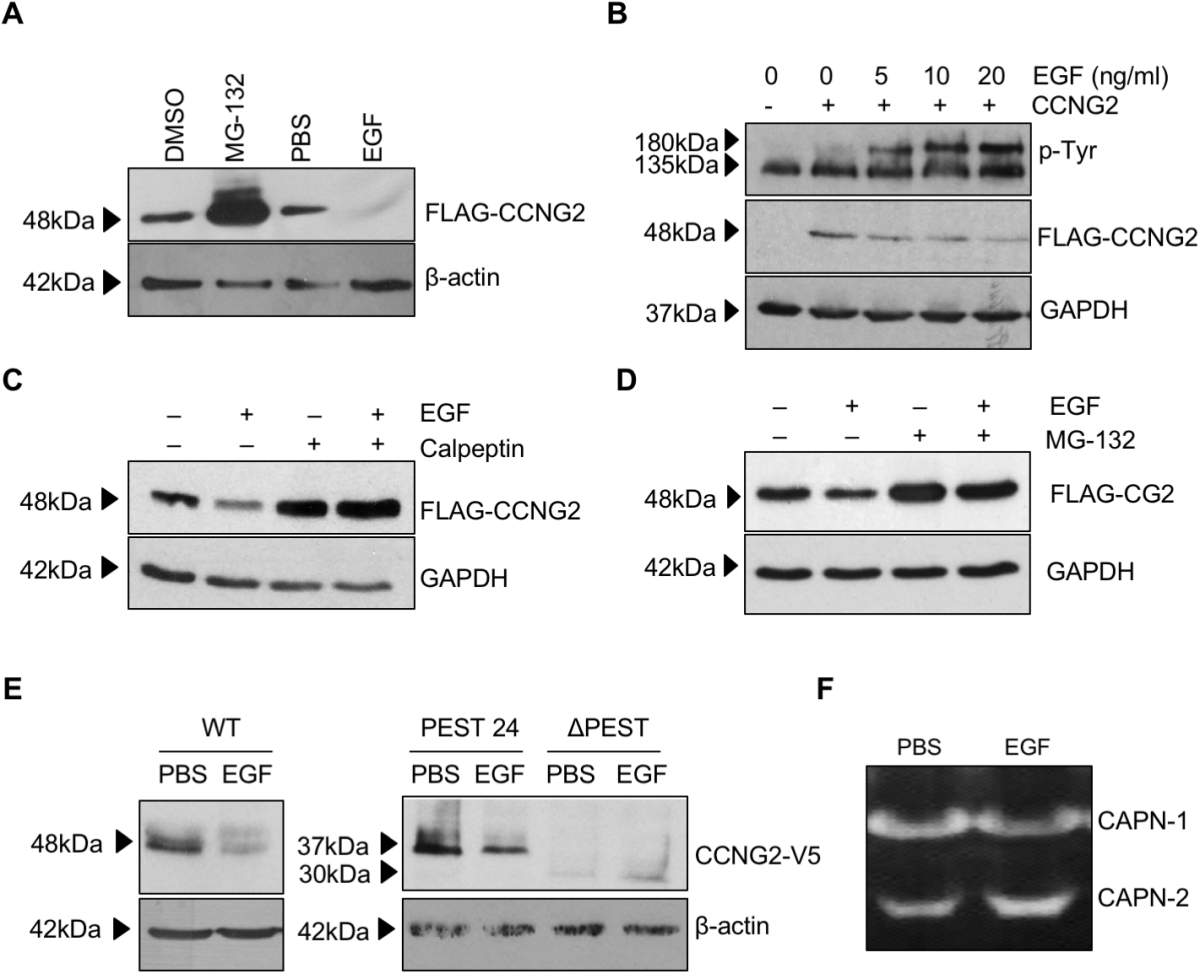
EGF enhances cyclin G2 degradation. A) OV2008 cells stably expressing cyclin G2 were treated with 10 μM MG-132, to block protein degradation, or 20ng/mL EGF, and their vehicle control for 1 hour, and chased with CHX to block translation for 2 hours. Treatment with MG-132 protected cyclin G2 from degradation, while treatment with EGF decreased cyclin G2 stability. B) ES2 cells were transiently transfected with a FLAG-CCNG2 construct or its vector control for 16h. The FLAG-CCNG2-transfected cells were then treated with CHX in the presence of various concentrations of EGF for 1h. Protein lysates were probed for anti-phospho-tyrosine (p-Tyr) and a band corresponding to the molecular size of EGFR was induced by EGF. A dose-dependent decrease of cyclin G2 stability was also observed. C) OV2008 cells were transiently transfected with FLAG-CCNG2 overnight and then treated with calpeptin (50 μM) for 30min, followed by EGF (20ng/ml) and CHX treatment for 2h. Inhibition of calpain blocked the effect of EGF on cyclin G2 degradation. D) SKOV3.ip1 transiently transfected with FLAG-CCNG2 were treated with or without EGF, in the presence or absence of MG-132. MG-132 attenuated the effect of EGF on cyclin G2 degradation. E) OV2008 cells were transfected with either full-length cyclin G2-V5 or PEST deletion mutants (PEST-24 or ΔPEST) and treated with EGF for 30 min before addition of CHX for another 2 hours. EGF increased degradation of the wild-type (WT) and PEST-24 cyclin G2, whereas removal of PEST domain abolished EGF-induced cyclin G2 degradation. F) OV2008 cells were treated with EGF (20 ng) or PBS as the control for 15 min prior to lysis. Casein zymography was performed to detect calpain 1 and calpain 2 activity. Clearing bands on the zamogram reveraled that EGF increased calpain 2, but not calpain 1, activity.

To further investigate the effect of EGFR signaling on cyclin G2 degradation, we used CHX to block *de novo* protein expression, and treated cells with EGF. As expected, in OV2008 cells, treatment with MG-132 increased, whereas treatment with EGF decreased, the level of cyclin G2 (Fig 4A). Similarly, in ES2 cells, treatment with EGF resulted in a dose-dependent decrease in cyclin G2 levels. Activation of EGFR, following EGF treatment, was confirmed using an anti-phospho-tyrosin antibody to detect the phosphorylated form of EGFR (Fig 4B). While treatment with EGF promoted degradation of cyclin G2, pretreatment with calpeptin (Fig 4C) or MG132 (Fig 4D) strongly abolished the effect of EGF. To test if the EGF-induced degradation depends on the PEST domain, OV2008 cells transfected with either the wild type, PEST 24 or ΔPEST deletion mutants were treated with or without EGF for 30mins and chased with CHX for 2 hours. EGF treatment decreased protein stability of both the wild-type and PEST24 cyclin G2 constructs, but did not affect the level of ΔPEST (Fig 4E). Finally, treatment with EGF resulted in an increase in calpain-2 activity, as revealed by casein zymography (Fig 4F).

## Discussion

We have previously demonstrated that cyclin G2 is a highly unstable protein and can be degraded via ubiquitin-proteosome pathway [28]. We also showed that the PEST domain is critically involved in the stability of cyclin G2. Since multiple degradative mechanisms can act on a single protein, and several studies have reported that PEST sequence can be targeted by calpain for degradation [34, 39], we investigated if cyclin G2 can be degraded by the calpain pathway. In the present study, we provided the first evidence that cyclin G2 instability is mediated, in part, by proteolytic cleavage of calpains and that the action of calpains is dependent on prior phosphorylation of cyclin G2, via the EGFR signaling pathway.

Several lines of evidence support the role of calpains in cyclin G2 degradation. First, *in vitro* studies showed that cell lysates incubated with calcium and purifed calpains had much lower cyclin G2 levels when compared with the control. The effect of calpain/calcium on cyclin G2 degradation was abolished by a calpain inhibitor, calpeptin. Second, *in vivo* studies revealed that treatment with A23187, which enhances the intracellular calcium concentration, reduced cyclin G2 stability, and this effect was reversed by protease inhibition. In addition, we observed an increase in cyclin G2 stability when cells were treated with a membrane permeable calpain inhibitor, calpeptin. Finally, knockdown of endogenous calpain-1 and calpain-2 expression by specific siRNAs resulted in an increase in cyclin G2 stability.

In this study, we observed that phosphorylation events are necessary for calpain-mediated cyclin G2 degradation. Pre-treatment of cell lysates with a phosphatase protected cyclin G2 from calpain-induced degradation. Similarly, studies in other systems reported that calpain action is induced by the phosphorylation of the target protein, and that inhibition of the kinases responsible for the phosphoryation, or mutation of the predicted phosphorylation site(s), is critical for calpain-mediated proteolysis [40, 41]. Therefore, it is possible that cyclin G2 is phosphorylated prior to calpain cleavage. Interestingly, screening of a kinase inhibitor library identified three inhibitors, AG1478, PP1 and PP2 that enhanced cyclin G2 stability. Although PP1 and PP2 are inhibitors of Src family kinases, they have also been reported to block the activity of EGFR [38]. We therefore focused on the EGFR pathway and showed that treatment with AG1478 not only increased cyclin G2 stability, but also completely abrogated the action of calpain on cyclin G2. On the other hand, EGF induced the degradation of cyclin G2.

**Fig 5:**
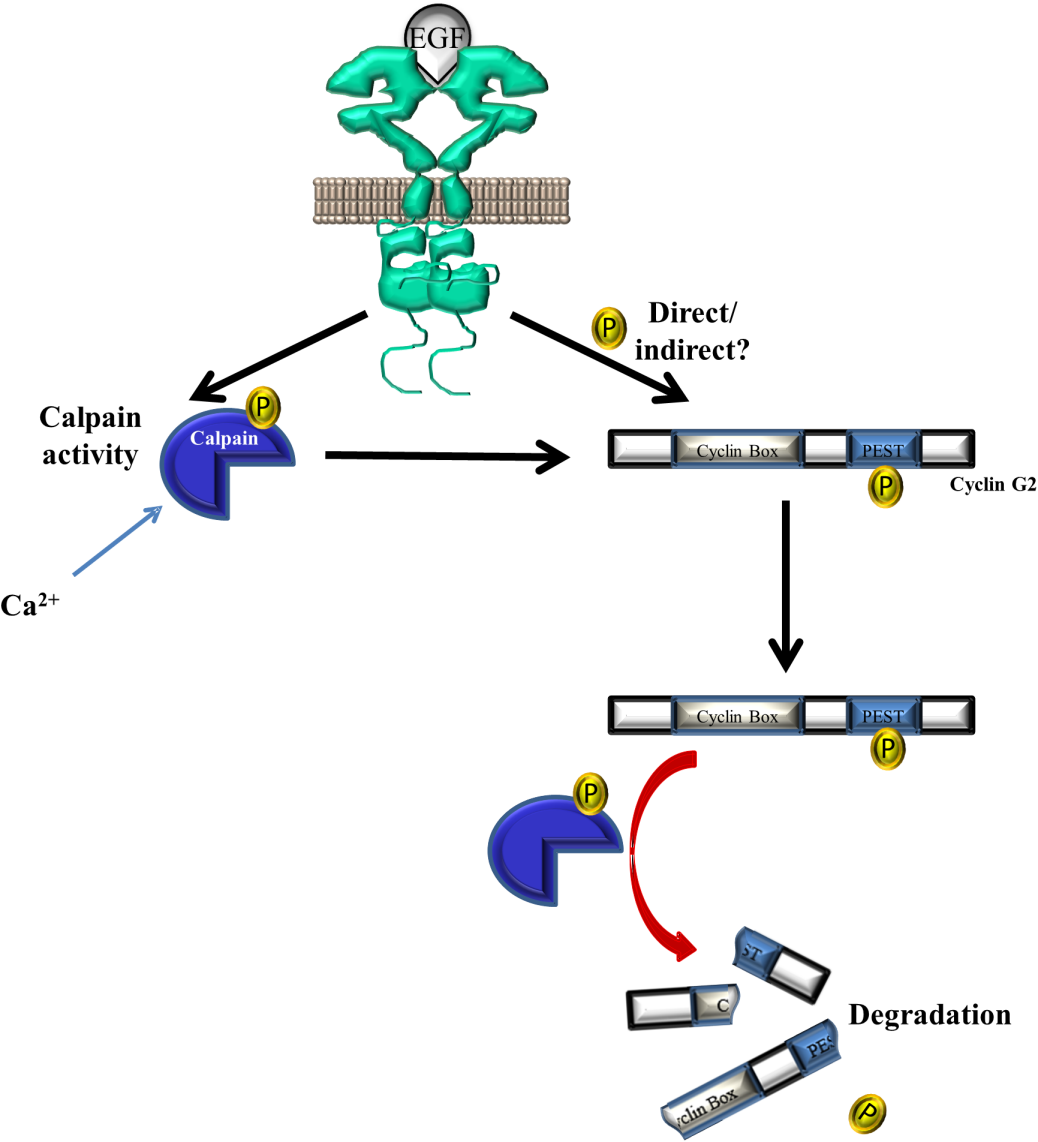
Proposed role of EGF in cyclin G2 degradation. EGF binds to its receptor, EGFR, to induce cyclin G2 phosphorylation, either directly or indirectly via downstream kinases. EGF may also activate calpain-2 to enhance its activity. Phosphorylated cyclin G2 is recognized and degraded by calpains.

Using casein zymography, we found that EGF-treatment promoted calpain 2 activity. This finding is consistent with several previous studies that reported EGF increased calpain-2 activity via the activation of ERK pathways [42–44]. Therefore, it is possible that EGFR activation, and downstream pathways, increase calpain 2 activity to enhance cyclin G2 degradation. In addition, EGFR may also phosphorylate cyclin G2, marking it for destruction by calpains and thereby increasing the interaction between calpain and cyclin G2. Interestingly, amino acids 272–294 of cyclin G2 share sequence similarity with an autophosphorylation motif on EGFR [9]. Bioinformatic tools predicted residues 284 and 285 as EGFR and Src phosphorylation sites. Therefore, EGFR may phosphorylate cyclin G2 directly, either via internalization with EGFR or by recruiting cyclin G2 to the plasma membrane. Additionally or alternatively, EGFR may activate downstream kinases to phosphorylate cyclin G2. It is well established that EGF actives diverse signaling cascades, including Src family kinases [45]. We propose that EGF induces cyclin G2 degradation via two mechanisms:(1) to increase calpain activity and (2) to induce cyclin G2 phosphorylation (Fig 5). Future studies are required to determine if EGFR phosphorylates cyclin G2 directly or indirectly.

Analyses of the cyclin G2 sequence revealed many potential phosphorylation sites. Surprisingly, treatment with inhibitors of CK-II, PKC, and CDK5, which have the highest predicted scores, did not affect cyclin G2 stability, even when used at very high concentrations. For the kinase inhibitor library screening, we only used a single dose (10 μM) and this may not be the effective dose for all inhibitors. Therefore, we cannot rule out the possibility that additional kinases are involved in regulating cyclin G2 stability. Future studies are needed to determine if additional kinases are involved in cyclin G2 phosphorylation and to confirm the precise role of phosphorylation in calpain-mediated degradation of cyclin G2.

The PEST domain is a polypeptide sequence enriched in proline, glutamic acid, serine and threonine and has been implicated in regulating the intrinsic stability of many proteins [46–48]. PEST domains have been regarded as recognition factors for many proteolytic proteins, including calpains, and protein kinases [47]. Phosphorylation at the PEST domain may also induce conformational changes elsewhere in the protein necessary for interaction with proteolytic components [47]. We have reported that the removal of the PEST domain greatly increased cyclin G2 stability [28]. In this study, we showed that both calpain and EGF treatment induced the degradation of the wild-type and PEST24 cyclin G2 constructs, but had no effect on the stability of cyclin G2 when the PEST domain was completely removed. Therefore, the first 24 amino acids of the PEST domain are critical for EGFR-induced degradation by calpain. Tyrosine 284 and 285, located in the PEST domain, are predicted to be phosphorylation sites for EGFR and Src kinases. Interestingly, both tyrosines are highly conserved across various species. This high degree of conservation suggests an important regulatory role of this site. Future studies will determine if mutagenesis of these potential EGFR/Src phosphorylation sites will abolish calpain-mediated cyclin G2 degradation. A calpain cleavage site is also predicted within the PEST domain. Whether calpains cleave at this site remains to be investigated.

One of the major limitations in studying cyclin G2 is the lack of reliable antibodies available for the detection of endogenous protein [28]. Future studies are required to confirm that the mechanism uncovered in this study reflects the regulation of the endogenously expressed protein.

In summary, data presented in this study has outlined a novel mechanism for degradation of cyclin G2. Firstly, the activation of EGFR may lead to the phosphorylation of cyclin G2 at the PEST domain, either directly or indirectly through downstream signaling pathways, such as Src kinases. This phosphorylation increases the susceptibility of cyclin G2 to degradation by calpains. Furthermore calpain-2 is activated by the EGFR pathway [42], and may also contribute to the increased degradation of cyclin G2. This pathway, in combination with other proteolytic mechanisms, including the 26S proteasome [28], may be responsible for the fast turnover of cyclin G2 and mediates the highly unstable nature of this protein. EGFR activation is a common contributor to malignancy, and many reports have implicated increased EGF signaling as a driver of both ovarian and cervical cancer [49–54]. Since we have also observed that cyclin G2 can exert potent anti-tumorigenic effects [18], it is possible that inhibition of cyclin G2 by EGFR is a significant event underlying the oncogenic actions of EGF and contributes to ovarian and cervical cancer development.

## Supporting information

S1 Table Kinase inhibitors tested and their effects on cyclin G2 stability. (DOCX)
