# Supplementary material for: Epidermal growth factor promotes cyclin G2 degradation via calpain-mediated proteolysis in gynaecological cancer cells
Source: PLoS One. 2017 Jun 22;12(6):e0179906. doi: 10.1371/journal.pone.0179906 (PMC5481008; doi:10.1371/journal.pone.0179906)
Supplement: S1 Table — (DOCX) [file pone.0179906.s001.docx]

Table S1. Effects of kinase inhibitors on cyclin G2 stability

| Chemical Names | Targets | Effects on cyclin G2 stability |
| --- | --- | --- |
| PD-98059 | MEK | No effect |
| U-0126 | MEK | No effect |
| SB-203580 | p38 MAPK | No effect |
| H-7 | PKA, PKG, MLCK, AND PKC | No effect |
| H-9 | PKA, PKG, MLCK, AND PKC | No effect |
| Staurosporine | Pan-specific | No effect |
| AG-494 | EGFRK, PDGFRK | No effect |
| AG-825 | HER1-2 | No effect |
| Lavendustin A | EGFRK | No effect |
| RG-14620 | EGFRK | No effect |
| Tyrphostin 25 | EGFRK | No effect |
| Tyrphostin 47 | EGFRK | No effect |
| Tyrphostin 51 | EGFRK | No effect |
| Tyrphostin 46 | EGFRK, PDGFRK | No effect |
| Tyrphostin 1 | Negative Control | No effect |
| Tyrphostin 23 | EGFRK | No effect |
| Tyrphostin AG 1478 | EGFRK | Increase |
| Tyrphostin AG 1288 | Tyrosine kinases | No effect |
| Tyrphostin AG 1295 | Tyrosine kinases | No effect |
| Tyrphostin 9 | PDGFRK | No effect |
| HNMPA | IRK | No effect |
| PKC-412 | PKC | No effect |
| Piceatannol | Srk | No effect |
| PP1 | Src family | Increase |
| PP2 | Src family | Increase |
| AG-490 | JAK-2 | No effect |
| AG-126 | IRAK | No effect |
| AG-370 | PDGFRK | No effect |
| AG-879 | NGFRK | No effect |
| Wortmannin | PI3-K | No effect |
| Y-27632 | Rock | No effect |
| ZM 449829 | JAK-3 | No effect |
| Rapamycin | mTor | No effect |
| Apigenin | CK-II | No effect |
| DRB | CK-II | No effect |
| GW 5074 | cRAF | No effect |
| SU1498 | Flk1 | No effect |
